# Survey on and analysis of the factors influencing the postoperative sleep quality of Chinese patients with oesophageal cancer

**DOI:** 10.1186/s12957-025-04008-5

**Published:** 2025-09-26

**Authors:** Xi-xi Yin, Xiao Yu, Yanyan Fang, Dandan Liu, Liping Yang, Li Liu, Yanhui Pan

**Affiliations:** 1 Nursing Department, Sun Yat-sen University Cancer Center, Kaiyang 5 Road, Huangpu District, Guang Zhou, Guangdong Province China; 2 School of Nursing, Guang Zhou Xin Hua University, No.19 Huamei Road, Longdong, Tianhe District, Guangdong Province Guang Zhou, China

**Keywords:** Oesophageal cancer, Sleep quality, Influencing factors, Postoperative sleep quality

## Abstract

**Background:**

Sleep quality problems are common in patients with cancer, and the likelihood of sleep disorders is high in postoperative patients. Patients with oesophageal cancer are prone to postoperative problems with sleep quality owing to the complexity of the surgery. Therefore, we aimed to understand sleep quality of patients after oesophageal cancer surgery, analyse the factors that influence sleep quality, and provide theoretical references to improve the sleep quality of patients after oesophageal cancer surgery.

**Methods:**

A self-designed general information questionnaire, the Pittsburgh Sleep Quality Index (PSQI) and the Hospital Anxiety and Depression Scale were used to conduct a questionnaire survey. This questionnaire was used to survey 119 patients who underwent oesophageal cancer surgery at our hospital's thoracic department from October 2020 to June 2021. Statistical methods such as Spearman correlation analysis and multiple regression analysis were used to analyse the sleep quality of the patients and explore the factors that influenced sleep quality.

**Results:**

(1) Among the 119 postoperative oesophageal cancer patients included in the study, 116 patients (97.48%) experienced sleep disturbance (PSQI≥7). The mean PSQI scores were 15.19±3.95; 60.5% (72/119) of patients experienced anxiety, and 48.74% (58/119) experienced depression. (2) Spearman correlation analysis revealed that patients' sleep quality scores negatively correlated with level of education and surgical approach (correlation coefficients of -0.23 and -0.27, respectively, *P*<0.05) and positively correlated with pain scores and nutritional risk (correlation coefficients of 0.26 and 0.17, respectively, *P*<0.05). The results revealed no correlation between anxiety or depression scores and PSQI scores. The average monthly household income was correlated with level of education, home residence, a burden of medical expenses, postoperative complications, and anxiety scores (correlation coefficients were 0.17, -0.28, -0.47, 0.26, and-0.24, respectively; *P*<0.05). The burden of medical expenses was also correlated with level of education and home residence (the correlation coefficients were -0.16 and 0.22, respectively;* P*<0.05). Postoperative complications were positively correlated with anxiety scores and depression scores (correlation coefficients were 0.34 and 0.27, respectively, *P*<0.05). (3) Multiple regression analysis revealed that surgical approach, pain scores, level of education, and nutritional risk scores affect the sleep quality of patients (95% CI=9.83–17.48, adjusted R^2^=0.23, *P*<0.05).

**Conclusions:**

The postoperative sleep quality of Chinese oesophageal cancer patients was generally poor, which was related to the surgical approach, education level, pain score, and nutritional risk score. Anxiety, depression scores, and average monthly household income may also indirectly affect sleep quality. These findings suggest that clinical caregivers should consider the above factors in the prevention and treatment of relevant symptoms to improve the sleep quality of patients.

## Background

Sleep is vital to health, and a good night’s sleep helps to clear the brain of metabolic waste, restore mental and physical energy, boost immunity, reduce the risk of chronic disease, and positively impact mental health [1]. Sleep quality refers to an individual’s satisfaction with the sleep experience and integrates several aspects such as sleep initiation, sleep maintenance, sleep quantity, and a feeling of being refreshed upon awakening [2]. Sleep disorders are manifestations of poor sleep quality [1, 3]. According to the latest China Sleep Research Report 2024, the incidence of insomnia among Chinese adults is as high as 38.2%, and the number of people with sleep disorders is as high as 510 million. Therefore, more than 500 million people in China struggle with sleep quality, making sleep quality a public social issue that seriously affects people’s quality of life and work [4]. Sleep disorders are also more common in cancer patients and are a risk factor for cancer [5]. Many cancer patients experience sleep disturbances after treatment, which may be related to surgery, chemical drug toxicity, and abnormal secretion of hormones [6–8].

Oesophageal cancer is a highly prevalent malignant tumour of the gastrointestinal tract worldwide, and its epidemiological characteristics have significant geographic variability. China has a high incidence of oesophageal cancer, accounting for more than 50% of the global incidence. The predominant form of oesophageal cancer in China is squamous cell carcinoma, which is closely related to diet (e.g., spicy food or pickled food), smoking, alcohol consumption, and genetic predispositions [9, 10]. Surgery is the main treatment for oesophageal cancer, but postoperative patients are often confronted with swallowing difficulties, malnutrition, pain, and psychological disorders, of deterioration of sleep quality has long been overlooked as a potential complication [11]. Studies [12, 13] have shown that sleep disorders not only exacerbate postoperative fatigue syndrome and immunosuppression but also may affect prognosis by mediating changes in the tumour microenvironment through inflammatory factors.

Although several studies [11, 14–17] have focused on sleep problems in cancer patients, few systematic investigations have been conducted in oesophageal cancer patients after surgery. The aim of this study was to assess the current status of sleep quality in Chinese postoperative oesophageal cancer patients (using the PSQI scale) through a cross-sectional survey, analyse the factors influencing sleep quality (e.g., pain, nutritional risk, psychological status and surgical modality), and explore the associations between sleep disorders and postoperative complications and quality of life. The results of this study may provide evidence for the development of targeted intervention strategies (e.g., stepped analgesia, psychological support and nutritional optimization) and lay the foundation for future exploration of the impact of sleep interventions on oncological prognosis.

## Methods

### Patients

Our study was a single-centre prospective observational cohort study. A convenience sampling method was used to administer a questionnaire to patients who had undergone oesophageal cancer surgery one week prior at the Department of Thoracic Surgery of Sun Yat-sen University Cancer Center from October 2020 to June 2021. The inclusion criteria were as follows: (1) were aged ≥ 18 years and had a clinical diagnosis of oesophageal occupancy or oesophageal mass with concurrent surgical treatment; (2) voluntarily participated in this study and provided informed consent; (3) had undergone surgery at least 7 days prior and been transferred to the general ward; and (4) had no history of sleep disorders or neuropsychiatric disorders. The exclusion criteria were as follows: (1) patients with preoperative complications such as asthma, chronic obstructive pulmonary disease (COPD), pneumonia, lung infection, or respiratory failure,; (2) patients with serious organic lesions or insufficiency of other organs such as the heart, brain, or kidney; patients with drowsiness and impaired consciousness after surgery; (3) patients with serious bleeding, injury, and severe intraoperative or postoperative complications during surgery; and (4) patients who were unable to answer questions due to serious complications such as postoperative nerve damage and respiratory failure.

All the subjects provided informed consent to participate in this study in accordance with the Declaration of Helsinki (Ethical Guideline: B2021-067-01).

### Measures

#### Patient information

Based on a literature review and the expertise of the investigators, we designed a questionnaire to collect basic patient information. The self-designed general information questionnaire included age, sex, education level, marital status, occupation, family income, payment method and health care burden, presence of hypertension and diabetes, smoking history, postoperative complications, and nutritional risk screening scores (NRS2002).

#### Measurement of sleep quality, anxiety and depression

We procured an instrument to evaluate sleep quality, the Pittsburgh Sleep Quality Index (PSQI). The PSQI [18] is a self-rated questionnaire that assesses sleep quality and disturbance over a 1-month time interval. The PSQI consists of 19 self-assessments and 5 questions related to the bed-partner or roommate (which are used for clinical information and do not impact the PSQI scores). The 19 self-assessment questions assess a wide variety of factors related to sleep quality, including estimates of factors related to sleep duration, which are grouped into seven component scores, each weighted equally on a 0–3 scale. The seven component scores are then summed to yield global PSQI scores, which range from 0 to 21; higher scores indicate worse sleep quality. The seven components of the PSQI are standardized versions of areas routinely assessed in clinical interviews of patients with sleep/wake complaints. These components are subjective sleep quality, sleep latency, sleep duration, habitual sleep efficiency, sleep disturbance, use of sleeping medications, and daytime dysfunction. The scoring of each component is illustrated in the Appendix. Subject instructions for the PSQI are contained in the text. Subjects can complete the entire index in 5–10 min, which can then be scored in 5 min. We used the Chinese version of the PSQI [19] to evaluate patients one week after surgery.

To investigate anxiety and depression in postoperative patients, we also procured an instrument, the HADS [20], to evaluate emotional Health of patients after undergoing oesophagectomy. The HADS examines anxiety and depression through two different subscales, each containing 7 items. The anxiety subscale is designed to detect generalized anxiety, whereas the depression subscale focuses on assessing pleasure deficit disorder. It has mainly been applied to screen for anxiety and depression in patients attending general hospitals. Sun et al. [21] retested the Chinese version of the HADS, and the Cronbach’s alpha coefficients for the overall HADS, anxiety subscale, and depression subscale were 0.879, 0.806, and 0.806, respectively, with good reliability and validity. The Chinese version of the HADS consists of 14 items, of which 7 items assess depression and 7 items assess anxiety. The scores of the two subscales of anxiety and depression are classified as 0–7 for asymptomatic patients, 8–10 for symptomatic patients, and 11–21 for symptomatic patients.

#### Outcome measures

The primary outcome metric focuses on the patient’s sleep quality scores, and the secondary outcome metrics are the patient’s general information profile, the occurrence of postoperative complications, pain score, nutritional risk score, and anxiety and depression scores. The secondary outcome metrics can be included in a multiple regression model as influences on sleep quality.

### Sample size

The statistical method used in our study was multiple linear regression. We used G-power (v3.1.9.2) software to calculate sample size [22] and assumed the following: α error problem = 0.05, power (1-β error problem) = 0.95, Tail(s) = two, and the number of predictors = 10. Using these methods and based on these assumptions, the minimum sample size was calculated to be ≥ 80 patients. Considering a 10%−20% sample loss rate, we calculated a sample size of no less than 96 patients.

### Data collection

This study was conducted by professionally trained investigators. The general information was collected by the investigators with the patients’ consent. The PSQI and HADS were administered by the investigator one week after the patient’s surgery to assess the patient’s sleep and emotional state during the one-week postoperative period. Investigators promptly addressed any questions from the patients, who needed approximately 5–10 min to complete the questionnaire.

### Date analysis

Data were analysed using IBM SPSS Statistics version 22.0 for Windows. Descriptive analysis was conducted to summarize the sociodemographic data, disease characteristics, and overall PSQI data of the study participants. The elements associated with PSQI scores were examined through several analytical techniques (such as Spearman and Kendall’s tau-b), depending on the nature of the data. Linear regression was used to predict the impact on PSQI score factors. All tests were two-sided, and *P* < 0.05 was considered statistically significant.

## Results

### Characteristics of the study patients

A total of 123 questionnaires were distributed. Four patients met the exclusion criteria and were unable to complete the questionnaire, including patients who experienced serious complications after surgery, three of whom had postoperative lung infection and one who experienced postoperative respiratory failure and was admitted to the intensive care unit. Ultimately, 119 patients completed all the questionnaires. As shown in Table 1, the average age of the participants was 62.71 ± 8.03 (range = 31–81) years, and the mean body mass index was 22.47 ± 3.14 (range = 15.54–31.51) kg/m^2^. Among these patients, 78.99% (94/119) were male, 26.89% (32/119) were not working, and 91.6% (109/119) had a burden of medical expenses; moreover, 92.4% (110/119) had squamous carcinoma, and 52.94% (63/119) underwent three-incision surgery with robot assistance/VATS, and the duration of surgery was 262.02 ± 136.41 min. The mean PSQI score was 15.19 ± 3.95 (range = 4–21), and 97.48% of patients (116/119) had sleep disturbances. Furthermore, 60.5% (72/119) experienced anxiety, 48.74% (58/119) experienced depression, and 92.43% were at nutritional risk. See Table 1 for details.

### Correlations between PSQI scores and other characteristics

We performed correlation factor analyses of general patient information, nutritional risk scores, pain scores, and anxiety and depression scores with PSQI scores, and the results revealed no correlations between anxiety and depression scores and PSQI scores. As shown in Table 2, the level of education, surgical approach, pain scores and nutritional risk scores were correlated with the PSQI score, whereas the level of education and surgical approach were negatively correlated with the PSQI score (correlation coefficients of −0.23 and − 0.27, respectively, *P* < 0.05). The pain and nutritional risk scores were positively correlated with the PSQI score (correlation coefficients of 0.26 and 0.17, respectively, *P* < 0.05). In addition, average monthly household income was correlated with the level of education, home residence, burden of medical expenses, postoperative complications, and anxiety scores (correlation coefficients were 0.17, −0.28, −0.47, 0.26, and − 0.24, respectively; *P* < 0.05). The burden of medical expenses was also correlated with the level of education and home residence (the correlation coefficients were − 0.16 and 0.22, respectively; *P* < 0.05). Postoperative complications were positively correlated with anxiety scores and depression scores (correlation coefficients were 0.34 and 0.27, respectively, *P* < 0.05). Anxiety scores were positively correlated with depression scores (correlation coefficient 0.68, *P* < 0.05). See Table 2 for details.

### Analysis of factors influencing sleep quality in patients with oesophageal cancer

Based on the results in Table 2, we assigned quantitative information to the factors that influence PSQI scores, including surgical approach and level of education, as detailed in Table 3. As shown in Table 4, the factors affecting the quality of sleep included surgical approach, pain score, level of education, and nutritional risk score (95% CI = 9.83–17.48, adjusted R^2^ = 0.23, *P* < 0.05). Multiple regression analyses revealed that pain and nutritional risk scores had positive effects on sleep quality scores (B values of 0.49 and 0.64, 95% CI = 0.18–0.81 and 0.08–1.20, respectively; *P* < 0.05). However, surgical approach and the level of education had a negative impact on sleep quality scores (B values of −0.94 and − 1.08, *95% CI=*−1.56-(−0.33) and − 1.82-(−0.35), respectively, *P* < 0.05).

**Table 1:** Characteristics of the study patients (*n* = 119)

Patient characteristic	Value($\:\stackrel{-}{x}$±s, *n*[%])	Patients’ characteristic	Value($\:\stackrel{-}{x}$±s, *n*[%])	Patient characteristic	Value($\:\stackrel{-}{x}$±s, *n*[%])
Age (years)	62.71 ± 8.03	Average monthly household income (RMB)		Whether postoperative complications occurred	
Body mass index (BMI kg/m^2^)	22.47 ± 3.14	<2000	23(19.33)	Yes	45(37.82)
Sex		2000–2999	14(11.76)	No	74(72.18)
Male	94(78.99)	3000–3999	25(21.01)	Surgery time	262.02 ± 136.41
Female	25(21.01)	4000–4999	10(8.40)	PSQI scores	15.19 ± 3.95
Employed		≥5000	47(39.50)	Sleep disturbances	
Yes	87(73.11)	Whether the patient was burdened by medical expenses		Yes (PSQI scores ≥ 7)	116(97.48)
No	32(26.89)	Yes	109(91.6)	No (PSQI scores<7)	3(2.52)
Level of education		No	10(8.4)	Anxiety scores	8.52 ± 3.68
Primary school or less	39(32.77)	Pathological type		Anxiety rating	
Middle school	46(38.66)	Squamous carcinoma	110(92.44)	Normal	47(39.50)
High school or secondary vocational school	29(24.37)	Adenocarcinoma	7(5.88)	Borderline anxiety	46(38.66)
Junior college and bachelor’s degree	4(3.36)	Other	2(1.68)	Clear anxiety	26(21.84)
Graduate degree	1(0.84)	Pathological staging		Depression scores	7.30 ± 3.72
Home residence		IA	23(19.33)	Depression rating	
Urban	26(21.85)	IB	38(31.93)	Normal	61(51.26)
Town	26(21.85)	II	11(9.24)	Borderline depression	35(29.41)
Countryside	67(56.30)	III	37(31.09)	Clear depression	23(19.33)
Smoking history		IV	10(8.41)	Nutritional risk scores	4.66 ± 1.17
Yes	82(68.91)	Surgical approach		Whether the patient was at nutritional risk	
No	37(31.09)	Three incisions with robot assistance/VATS	63(52.94)	Yes	110(92.43)
Drinking history		Two incisions	18(15.13)	No	9(7.57)
Yes	68(57.14)	One incision	38(31.93)		
No	51(43.86)	Pain	6.15 ± 2.06		

*BMI * Weight/height^2^ body mass index, *one incision * Radical oesophageal cancer surgery via the left/right chest, *two incisions * Radical oesophageal cancer surgery via an open incision in the chest and epigastrium, *three incisions * Radical oesophageal cancer surgery via an open incision in the neck, chest and epigastrium, *VATS * Laparoscopic, postoperative complications including tracheal fistulas, coeliac chest, and gastroesophageal reflux

**Table 2:** Correlations between PSQI scores and other characteristics. (*n* = 119)

Projects	PSQI scores	Level of education	Home residence	Average monthly household income	Whether the patient was burdened with medical expenses	Surgical approach	Whether postoperative complications occurred	Anxiety scores	Depression scores	Pain
Level of education	−0.23*									
Home residence	0.04	−0.34								
Average monthly household income	0.04	0.17*	−0.28*							
Whether the patient was burdened by medical expenses	0.01	−0.16*	0.22**	−0.47**						
Surgical approach	−0.27**	−0.06	0.11	0.00	−0.01					
Whether postoperative complications occurred	−0.03	0.00	0.10	−0.18**	0.08	−0.06				
Anxiety scores	0.09	−0.05	0.14	−0.24**	0.26**	0.03	0.34**			
Depression scores	−0.04	−0.11	0.11	−0.13	0.15	0.01	0.27**	0.68**		
Pain	0.26**	−0.02	0.00	0.01	0.05	0.08	0.00	0.02	−0.07	
Nutritional risk scores	0.17*	0.06	−0.14	0.08	−0.08	−0.04	−0.11	−0.04	−0.17	0.04

*=*P* < 0.05, **=*P* < 0.01

**Table 3:** Including factor assignment in linear regression

Independent	Reassignment
Level of education	Primary school and below = 1; middle school = 2; high school or secondary vocational school = 3; junior college and bachelor’s degree = 4; graduate degree = 5
Surgical approach	three incisions with robot assistance = 1; three incisions with VATS = 2; two incisions = 3; radical surgery for oesophageal carcinoma =4

One incision open heart surgery, two incisions open incisions on the chest and upper abdomen, three incisions open incisions on the neck, chest, upper abdomen, *VATS* Video-assisted thoracic surgery

**Table 4:** Analysis of factors influencing sleep quality in patients with oesophageal cancer

Predictor variable	B value	S.E.	β	T	95% CI	*R*²	Adjust *R*²
Constant	13.65	1.93	-	7.07	9.83–17.48	0.48	0.23
Surgical approach	−0.94	0.31	−0.25^*^	−3.03	−1.56-(−0.33)		
Pain	0.49	0.16	0.26^*^	3.13	0.18–0.81		
Level of education	−1.08	0.37	−0.24^*^	−2.93	−1.82-(−0.35)		
Nutritional risk scores	0.64	0.28	0.19^*^	2.25	0.08–1.20		

*CI * Confidence interval

*=*P* < 0.05

## Discussion

### Prevalence and severity of postoperative sleep disorders in oesophageal cancer patients

In this study, the incidence of sleep disorders in postoperative oesophageal cancer patients was as high as 97.49%, with a mean PSQI score of 15.19 ± 3.95, which was significantly greater than the threshold for the healthy population (≥ 7). This incidence of sleep disorders was also greater than the incidence of sleep disorders reported in other studies, which ranged from 50.8% to 73.5% [17, 24–28]. This difference may be related to the different durations of the investigation (we investigated the postoperative period), the long average intraoperative time (mean of 262.02 min), and the complexity of the surgical approach (52.94% of the surgeries being the three-incision procedure (open incision radical oesophageal cancer surgery of the neck, chest, and epigastrium)). The meta-analysis by Mohammed et al. [29] revealed that the prevalence of sleep disorders in cancer patients ranged from 15.3% to 99.8% [30–32], which may be related to differences in the cut-off values of the scales between studies.

This result suggests that sleep quality is generally impaired in oesophageal cancer patients after surgery, a finding that is consistent with the study by Clevenger et al. [12], who studied the negative impact of surgical trauma, pain and psychological stress on sleep. Notably, sleep disturbances were positively correlated with the incidence of postoperative complications (e.g., anastomotic fistula and lung infection) (*P* < 0.05), which may further disrupt sleep rhythms by prolonging the recovery period and exacerbating physiological discomfort due to complications [28].

### ‌Analysis of factors influencing postoperative sleep quality

Among the findings (Table 4), the factors affecting the quality of sleep included surgical approach, pain score, level of education, and nutritional risk score (95% CI = 9.83–17.48, adjusted R^2^ = 0.23, *P* < 0.05). The anxiety and depression scores did not influence sleep quality, a result that was slightly unexpected. Clevenger et al. [12] and James et al. [41] reported that psychological stress has a negative effect on sleep quality. The reasons for this difference in results may be related to the differences in the survey scales and the number of people included. However, we also found that many postoperative oesophageal cancer patients still suffer from anxiety (60.5%) and depression (48.74%). Therefore, we will explore the possible impact of anxiety and depression scores on sleep quality in the following discussion.

First, pain and nutritional risk scores had positive effects on sleep quality scores (B values of 0.49 and 0.64, 95% CI = 0.18–0.81 and 0.08–1.20, respectively, *P* < 0.05). Thus, higher pain scores and higher nutritional risk screening scores were associated with higher sleep quality scores, indicating potentially poorer sleep quality. Yao et al. [32] similarly reported elevated postoperative pain scores in patients with suboptimal sleep (PSQI ≥ 6), with 47% of women needing additional analgesics. Nociception-induced sympathetic activation may increase nocturnal arousal frequency, whereas interindividual variability in nonsteroidal anti-inflammatory drug (NSAID) sensitivity partially explains limitations in pain management [34]. We may need to develop stepped analgesic regimens using visual analogue scoring (VAS) in combination with drug half-life and timed administration, for example, in combination with nonpharmacological interventions (e.g., music therapy) to reduce drug dependence [35]. Interestingly, nutritional risk scores were positively correlated with sleep quality scores, and nutritional risk scores (92.43% of patients were at risk) were associated with hypoproteinaemia and anaemia, which may exacerbate the vicious circle of fatigue–insomnia by affecting neurotransmitter (e.g., 5-hydroxytryptamine) synthesis [36]. Therefore, we may need to focus on improving the nutritional status of our patients during the preoperative period.

Second, we found that the surgical approach and level of education had a negative impact on sleep quality scores (B values were − 0.94 and − 1.08, *95% CI=*−1.56-(−0.33) and − 1.82-(−0.35), respectively, *P* < 0.05). Specifically, a simpler surgical approach and higher the education level corresponded to a lower the sleep quality score of the patient, indicating that the patient had better sleep quality. This finding may be related to the fact that one incision (surgical approach) has a less negative impact on sleep quality than do three incisions with robot assistance/VATS. Furthermore, minimally invasive surgery (e.g., thoracoscopy) had a less negative impact on sleep quality than did open surgery, which may be related to its less traumatic nature and quicker recovery. Song et al. [37] reported that thoracoscopic surgery improves the quality of sleep by decreasing intercostal nerve injury and the stress response and by lowering postoperative pain and the levels of inflammatory factors, such as IL-6 and HMGB-1. In our study, patients with a higher level of education (*P* < 0.05) were motivated to learn about surgery-related content, were more likely to receive information about postoperative rehabilitation, and may have been able to reduce medication dependence and improve sleep quality by proactively engaging in a rehabilitation programme (e.g., adjusting the sleep environment (use of earplugs and eye masks) and employing nonpharmacological analgesia (e.g., music therapy) [38].

Finally, the results of the correlation analysis (Table 2) revealed that average monthly household income, being burdened by medical expenses, postoperative complications, and anxiety and depression scores were not correlated with PSQI scores, but these factors were correlated with each other. For example, anxiety and depression scores were positively correlated with postoperative complications (correlation coefficients were 0.34 and 0.27, respectively, *P* < 0.05), i.e., the occurrence of postoperative complications may increase the anxiety and depression status of patients. The average monthly household income was also associated with postoperative complications, medical burden, and anxiety (the correlation coefficients were − 0.18, −0.47, and − 0.24, respectively, *P* < 0.05). This finding indicates that the poorer the patient’s financial condition is, the less able they are to afford medical care for possible complications, and the more likely they are to experience anxiety and depression (anxiety scores were positively correlated with depression scores in our study). Together, these factors may constitute a source of psychological stress, which is similar to the findings of Xiao et al. [39], who reported that the three-day postoperative depression score was the best predictor of insomnia (AUC = 0.818). Additionally, Sato et al. [40] reported that patients with financial difficulties who could not afford a multimodal analgesic regimen had significantly higher postoperative VAS scores than did the control group (6.2 vs. 4.5, *P* = 0.02), as well as a 1.8-fold increase in the incidence of sleep disturbance. Therefore, for patients with financial difficulties, health insurance policies and social charitable resources can be integrated [40] to consider both patient’s financial situation and psychological status; a study by James et al. [41] also suggested that the use of cognitive behavioural therapy (CBT) can be targeted to alleviate the interaction between anxiety and sleep disorders.

## Conclusion‌

In this study, we investigated the postoperative sleep quality of Chinese patients with oesophageal cancer and found that postoperative sleep disorders were prevalent in oesophageal cancer patients. The main factors influencing sleep quality were level of education, surgical approach, pain score and nutritional risk score. Anxiety, depression, and average monthly household income may also indirectly affect sleep quality, which suggests that we should consider the influence of these factors in the postoperative period and intervene in a timely manner to improve the psychological condition of patients and provide appropriate health education.

### ‌Limitations

The cross-sectional design of this study makes elucidating the dynamic changes in sleep disorders among patients with oesophageal cancer difficult. Therefore, longitudinal follow-up combined with polysomnography (PSG) is needed in the future. In addition, the impact of cultural differences (e.g., the family care model) on sleep quality was not included and needs to be further explored in subsequent studies.

## Data Availability

No datasets were generated or analysed during the current study.

## References

[1] Palagini L, Hertenstein E, Riemann D, Nissen C. Sleep, insomnia and mental health. J Sleep Res. 2022;31(4):e13628. 10.1111/jsr.13628. 35506356 10.1111/jsr.13628

[2] Cappuccio FP, D’Elia L, Strazzullo P, Miller MA. Quantity and quality of sleep and incidence of type 2 diabetes: a systematic review and meta-analysis. Diabetes Care. 2010;33:414–20. 19910503 10.2337/dc09-1124 PMC2809295

[3] K Pavlova M, Latreille V. Sleep disorders. Am J Med. 2019;132(3):292–9. 10.1016/j.amjmed.2018.09.021. 30292731 10.1016/j.amjmed.2018.09.021

[4] JX Wang, Y Zhang, N Liu. China Sleep Research Report [M].China:Social Sciences Academic Press [2025-01-15]. 2024.

[5] Mogavero MP, DelRosso LM, Fanfulla F, Bruni O, Ferri R. Sleep disorders and cancer: state of the art and future perspectives. Sleep Med Rev. 2021;56:101409. 10.1016/j.smrv.2020.101409. 33333427 10.1016/j.smrv.2020.101409

[6] Walker W, Borniger JC. Molecular mechanisms of Cancer-Induced sleep Disruption[J]. Int J Mol Sci. 2019;20(11):2780. 10.20944/preprints201905.0040.v1. 31174326 10.3390/ijms20112780 PMC6600154

[7] He C, He Y, Yang T, et al. Relationship of sleep-quality and social-anxiety in patients with breast cancer: a network analysis. BMC Psychiatry. 2023;23(1):887. 10.1186/s12888-023-05262-1. Published 2023 Nov 28. 38017507 10.1186/s12888-023-05262-1 PMC10683122

[8] Vin-Raviv N, Akinyemiju TF, Galea S, Bovbjerg DH. Sleep disorder diagnoses and clinical outcomes among hospitalized breast cancer patients: a nationwide inpatient sample study. Support Care Cancer. 2018;26(6):1833–40. 10.1007/s00520-017-4012-1. 29264658 10.1007/s00520-017-4012-1

[9] Han B, Zheng R, Zeng H, et al. Cancer incidence and mortality in China, 2022. J Natl Cancer Center. 2024;4(1):47–53. 10.1016/j.jncc.2024.01.006. 39036382 10.1016/j.jncc.2024.01.006 PMC11256708

[10] Bray F, Laversanne M, Sung H, et al. Global cancer statistics 2022: GLOBOCAN estimates of incidence and mortality worldwide for 36 cancers in 185 countries. CA Cancer J Clin. 2024;74(3):229–63. 10.3322/caac.21834. 38572751 10.3322/caac.21834

[11] Schandl A, Johar A, Anandavadivelan P, Vikström K, Mälberg K, Lagergren P. Patient-reported outcomes 1 year after oesophageal cancer surgery. Acta Oncol. 2020;59(6):613–9. 10.1080/0284186X.2020.1741677. 32193960 10.1080/0284186X.2020.1741677

[12] Clevenger L, Schrepf A, Christensen D, et al. Sleep disturbance, cytokines, and fatigue in women with ovarian cancer. Brain Behav Immun. 2012;26(7):1037–44. 10.1016/j.bbi.2012.04.003. 22543257 10.1016/j.bbi.2012.04.003 PMC3434312

[13] Zhao C, Grubbs A, Barber EL. Sleep and gynecological cancer outcomes: opportunities to improve quality of life and survival. Int J Gynecol Cancer. 2022;32(5):669–75. 10.1136/ijgc-2022-003404. 35331996 10.1136/ijgc-2022-003404 PMC9064983

[14] Lagergren P, Johar A, Rosenlund H, et al. Severe reflux, sleep disturbances, and health-related quality of life after esophageal cancer surgery. J Cancer Surviv. 2021;15(6):818–24. 10.1007/s11764-020-00974-9. 33502722 10.1007/s11764-020-00974-9 PMC8519838

[15] Schandl A, Färnqvist K, Mälberg K, Nielsen S, Lagergren P. Self-care advice for patients after surgery for oesophageal cancer - a mixed-methods systematic review. J Cancer Surviv. 2024. 10.1007/s11764-024-01551-0. 38361104 10.1007/s11764-024-01551-0 PMC12283782

[16] Scarpa M, Pinto E, Saadeh LM, et al. Sleep disturbances and quality of life in postoperative management after esophagectomy for esophageal cancer. World J Surg Oncol. 2014;12(1):156. 10.1186/1477-7819-12-156. 24886219 10.1186/1477-7819-12-156 PMC4032352

[17] Maroufi N, Sohrabi M, Mehrabadi S, et al. Poor sleep quality and its influencing factors among Iranian patients with esophageal and gastric cancer. Middle East J Dig Dis. 2024;16(1):39–46. 10.34172/mejdd.2024.367. 39050101 10.34172/mejdd.2024.367 PMC11264832

[18] Buysse DJ, Reynolds CF 3rd, Monk TH, Berman SR, Kupfer DJ. The Pittsburgh sleep quality index: a new instrument for psychiatric practice and research. Psychiatry Res. 1989;28(2):193–213. 10.1016/0165-1781(89)90047-4. 10.1016/0165-1781(89)90047-4 2748771

[19] Xianchen LIU. TANG maoqin.a study on the reliability and validity of the Pittsburgh sleep quality Index[J]. Chin J Psychiatry. 1996;29(2):5. 10.1007/BF02951625.

[20] Zigmond AS, Snaith RP. The hospital anxiety and depression scale. Acta Psychiatr Scand. 1983;67(6):361–70. 6880820 10.1111/j.1600-0447.1983.tb09716.x

[21] Zhenxiao SUN, HuaXue LIU, Linying JIAO et al. A study on the reliability and validity of the hospital anxiety and depression Scale[J]. Chin J Clin Physicians:Electronic Ed, 2017(2):4. 10.3877/cma.j.issn.1674-0785.2017.02.005

[22] Al-Harbi E J H .Sample Size in Multiple Regression Models: A simulation study[J]. Journal of Namibian Studies. 2023;(34):1661.

[23] Wang JP, Lu SF, Guo LN, Ren CG, Zhang ZW. Poor preoperative sleep quality is a risk factor for severe postoperative pain after breast cancer surgery: a prospective cohort study. Medicine (Baltimore). 2019;98(44):e17708. 10.1097/MD.0000000000017708. 31689803 10.1097/MD.0000000000017708 PMC6946447

[24] Mystakidou K, Parpa E, Tsilika E, et al. Sleep quality in advanced cancer patients. J Psychosom Res. 2007;62(5):527–33. 10.1016/j.jpsychores.2006.11.008. 17467407 10.1016/j.jpsychores.2006.11.008

[25] George GC, Iwuanyanwu EC, Anderson KO, et al. Sleep quality and its association with fatigue, symptom burden, and mood in patients with advanced cancer in a clinic for early-phase oncology clinical trials. Cancer. 2016;122(21):3401–9. 10.1002/cncr.30182. 27412379 10.1002/cncr.30182

[26] Momayyezi M, Fallahzadeh H, Farzaneh F, Momayyezi M. Sleep quality and cancer-related fatigue in patients with cancer. J Caring Sci. 2021;10(3):145–52. 10.34172/jcs.2021.021. (**Published 2021 Aug 23**). 34849358 10.34172/jcs.2021.021 PMC8609120

[27] Shorofi SA, Nozari-Mirarkolaei F, Arbon P, Bagheri-Nesamie M. Depression and sleep quality among Iranian women with breast cancer. Asian Pac J Cancer Prev. 2021;22(11):3433–40. 10.31557/APJCP.2021.22.11.3433. Published 2021 Nov 1. 34837896 10.31557/APJCP.2021.22.11.3433 PMC9068183

[28] Memtsoudis S, Liu SS, Ma Y, et al. Perioperative pulmonary outcomes in patients with sleep apnea after noncardiac surgery. Anesth Analg. 2011;112(1):113–21. 10.1213/ANE.0b013e3182009abf. 21081775 10.1213/ANE.0b013e3182009abf

[29] Al Maqbali M, Al Sinani M, Alsayed A, Gleason AM. Prevalence of sleep disturbance in patients with cancer: a systematic review and meta-analysis. Clin Nurs Res. 2022;31(6):1107–23. 10.1177/10547738221092146. 35484919 10.1177/10547738221092146 PMC9266067

[30] Mercadante S, Adile C, Ferrera P, Masedu F, Valenti M, Aielli F. Sleep disturbances in advanced cancer patients admitted to a supportive/palliative care unit. Support Care Cancer. 2017;25(4):1301–6. 10.1007/s00520-016-3524-4. 27957622 10.1007/s00520-016-3524-4

[31] Fong TCT, Ho RTH. Mindfulness facets predict quality of life and sleep disturbance via physical and emotional distresses in Chinese cancer patients: a moderated mediation analysis. Psychooncology. 2020;29(5):894–901. 10.1002/pon.5363. 32065693 10.1002/pon.5363

[32] Chan YN, Jheng YW, Wang YJ. Chemotherapy-induced peripheral neurotoxicity as a risk factor for poor sleep quality in breast cancer survivors treated with docetaxel. Asia Pac J Oncol Nurs. 2020;8(1):68–73. 10.4103/apjon.apjon_51_20. 33426192 10.4103/apjon.apjon_51_20 PMC7785078

[33] Yao ZW, Zhao BC, Yang X, Lei SH, Jiang YM, Liu KX. Relationships of sleep disturbance, intestinal microbiota, and postoperative pain in breast cancer patients: a prospective observational study. Sleep Breath. 2021;25(3):1655–64. 10.1007/s11325-020-02246-3. 33211236 10.1007/s11325-020-02246-3 PMC8376716

[34] Stehlik R, Ulfberg J, Zou D, Hedner J, Grote L. Perceived sleep deficit is a strong predictor of RLS in multisite pain - A population based study in middle aged females. Scand J Pain. 2017;17:1–7. 10.1016/j.sjpain.2017.06.003. 28850361 10.1016/j.sjpain.2017.06.003

[35] Wurm WE, Lechner A, Schmidt R, et al. Optimierung der schmerztherapie an neurologischen stationen [Optimising pain therapy for neurological inpatients]. Fortschr Neurol Psychiatr. 2015;83(3):149–56. 10.1055/s-0034-1399111. 25794320 10.1055/s-0034-1399111

[36] Yoerizta Ratu AN. Arina Widya murni. Risk factors for sleep disorders in patients with chronic pain: A Meta-Analysis. Bioscientia Medicina: J Biomed Translational Res. 2024;8(10):5172–84. 10.37275/bsm.v8i10.1100.

[37] Song B, Chang Y, Li Y, Zhu J. Effects of transcutaneous electrical acupoint stimulation on the postoperative sleep quality and pain of patients after Video-Assisted thoracoscopic surgery: A prospective, randomized controlled trial. Nat Sci Sleep. 2020;12:809–19. 10.2147/NSS.S270739. 33154688 10.2147/NSS.S270739 PMC7606945

[38] Da Silva FR, Guerreiro RC, Barreto AT, Brant VM, Silva A, De-Mello MT. Can Improving Postoperative Sleep Speed Up Surgical Recovery? [published correction appears in Sleep Sci. 2024;17(3):e1-e2. doi: 10.1055/s-0044-1791698.]. Sleep Sci. 2024;17(3):e335-e338. 10.1055/s-0044-1785522 10.1055/s-0044-1785522 PMC11390163 39268341

[39] Huang X, Wu D, Wu AS, Wei CW, Gao JD. The association of insomnia with depression and anxiety symptoms in patients undergoing noncardiac surgery. Neuropsychiatr Dis Treat. 2021;17:915–24. 10.2147/NDT.S296986. 33790560 10.2147/NDT.S296986 PMC8008159

[40] Sato D, Yoshinaga N, Nagai E, Hanaoka H, Sato Y, Shimizu E. Randomised controlled trial on the effect of internet-delivered computerised cognitive-behavioural therapy on patients with insomnia who remain symptomatic following hypnotics: a study protocol. BMJ Open. 2018;8(1):e018220. 10.1136/bmjopen-2017-018220. Published 2018 Jan 30. 29382675 10.1136/bmjopen-2017-018220 PMC5829590

[41] James AC, Reardon T, Soler A, James G, Creswell C. Cognitive behavioural therapy for anxiety disorders in children and adolescents. Cochrane Database Syst Rev. 2020;11(11):CD013162. 10.1002/14651858.CD013162.pub2. 33196111 10.1002/14651858.CD013162.pub2 PMC8092480

